# Maternal attachment security modulates the relationship between vulnerability to anxiety and attentional bias to threat in healthy children

**DOI:** 10.1038/s41598-024-55542-3

**Published:** 2024-03-12

**Authors:** Catherine Raymond, Rebecca Cernik, Myriam Beaudin, Maryse Arcand, Florence Pichette, Marie-France Marin

**Affiliations:** 1 Department of Psychology, Université du Québec À Montréal, Montreal, Canada; 2 Stress, Trauma, Emotion, Anxiety, and Memory (STEAM) Lab, Research Centre of the Institut Universitaire en Santé Mentale de Montréal, 7331 Hochelaga, Montreal, QC H1N 3V2 Canada; 3 Department of Psychiatry and Addictology, Faculty of Medicine, University of Montreal, Montreal, Canada

**Keywords:** Attentional bias to threat, Visual search task, Youth, Vulnerability to anxiety, Parent–child security, Psychology, Human behaviour, Cognitive neuroscience

## Abstract

This study aimed to investigate whether attentional bias to threat, commonly observed in clinically anxious children, also manifests in healthy children, potentially aiding the early detection of at-risk individuals. Additionally, it sought to explore the moderating role of parent–child attachment security on the association between vulnerability factors (anxiety sensitivity, intolerance of uncertainty, perseverative cognitions) as indicators of vulnerability to anxiety, and attentional bias towards threat in healthy children. A total of 95 children aged 8 to 12 years completed the Visual Search Task to assess attentional bias. Vulnerability to anxiety was measured using a composite score derived from the Childhood Anxiety Sensitivity Index, Intolerance of Uncertainty Scale for Children, and Perseverative Thinking Questionnaire. Parent–child attachment security was assessed using the Security Scale-Child Self-Report. Analyses revealed that higher vulnerability to anxiety was associated with faster detection of anger-related stimuli compared to neutral ones, and this association was further influenced by high maternal security. These findings in healthy children suggest an interaction between specific factors related to anxiety vulnerability and the security of the mother–child relationship, leading to cognitive patterns resembling those seen in clinically anxious individuals. These results hold promise for early identification of children at risk of developing anxiety disorders.

## Introduction

Anxiety disorders are among the most frequently diagnosed psychopathologies in childhood, with an incidence that notably increases after the onset of puberty^1,2^. Given that the emergence of anxiety disorders in youth represents an important factor in the chronicity and severity of the disorder^3^, it is crucial to expand our understanding of the etiology of anxiety in youth. In the long term, this could lead to better early identification of children at risk of developing an anxiety disorder. According to cognitive theories of anxiety (for a review, see^4^), attentional bias to threat plays a critical role in the etiology/maintenance of anxiety disorders in children and adults.

In healthy individuals, attentional bias to threat allows them to detect and respond to potential dangers more quickly and thus, is believed to be highly adaptive from an evolutionary point of view^5^. Two distinct attentional processes can be analyzed experimentally through cognitive tasks^6^: (1) attentional engagement towards threat and (2) attentional disengagement from threat. Attentional engagement towards threat, often arising from amygdala hyperactivity^7^, is evidenced by a reduced threshold for detecting threatening over neutral or positive stimuli^8^. This results in initial hypervigilance that enhances early threat detection and may subsequently elevate anxiety levels^9^. Attentional disengagement from threat is an automatic process that allows shifting attention away from threatening stimuli, occurring rapidly and outside conscious awareness^10^. A wealth of studies have demonstrated that attentional bias to threat is exacerbated in adults suffering from anxiety disorders and individuals presenting elevated levels of trait anxiety (for a meta-analysis and review on the topic, see^8^). In support of this body of literature and according to these authors, more than 11,000 studies would have to find an absence of an association between the two constructs to doubt the involvement of attentional bias to threat in clinical anxiety^8^.

Although research on attentional bias to threat is extensive in adults, results are inconsistent in children^8^. On one hand, there is evidence for accentuated attentional bias to angry faces in various forms of pediatric anxiety disorders (generalized anxiety disorder, separation anxiety, and social phobias) compared to healthy populations (for a review, see^11^). On the other hand, a few studies have failed to show a significant difference in attentional bias in children with anxiety disorders compared to their healthy peers (for a meta-analysis, see^12^). Contributing to these discrepancies, the age of participants is a pivotal factor, as it is associated with the ongoing development of key brain regions involved in threat detection and regulation, such as the amygdala and the prefrontal cortex, which are not yet fully mature^13^. These discrepant results could also be due to the variety of psychopathologies studied in association with an attentional bias to threat and a failure to examine both attentional processes (engagement and disengagement). Finally, the lack of studies in healthy youth may have concealed the attentional bias to threat continuum, as well as the markers of vulnerability associated with it. Identifying attentional bias patterns among healthy children with heightened vulnerability to later develop clinical anxiety could be a first step towards better understanding one of the potential mechanisms involved in the course of the psychopathology.

Research has identified several factors, present to varying degrees in the general population, that contribute to the vulnerability for developing anxiety disorders in youth^14–20^. These vulnerability factors include (1) anxiety sensitivity^21^, (2) intolerance to uncertainty^22^, (3) perseverative cognitions^23^, and (4) trait anxiety^18^, all of which play a pivotal role in shaping the susceptibility to anxiety disorders. In a recent longitudinal study in healthy youth (9–14 years old) during the COVID-19 pandemic, we showed that a greater *vulnerability to anxiety* (assessed using a composite score of these factors) predicted higher psychological distress over one year^24^. Although these vulnerability factors are known to predispose to psychopathology, studies have failed to document their cognitive correlates in a healthy population that expresses these traits to varying degrees. Yet, higher expression of vulnerability to anxiety in healthy individuals may be associated with attentional biases similar to those observed in children with clinical anxiety. In the long term, this could lead to better early identification of children at risk of developing an anxiety disorder.

Moreover, an important factor to consider when studying the association between vulnerability to anxiety and attentional bias is child attachment security. Indeed, when a child is exposed to a threatening situation, the attachment system is activated to promote security-seeking behaviors^25^. Children with an insecure attachment with the mother or both parents may have a heightened sense of insecurity and uncertainty about the availability and responsiveness of their attachment figures and in turn, lead to increased vigilance for potential threats in their environment^26^. As such, it was shown that children with insecure attachment styles towards their mothers present exacerbated attentional bias to threat during an experimental paradigm^27^. Moreover, insecure attachment styles were associated with elevated anxiety symptoms in healthy children^28,29^ and predicted the onset of anxiety disorders in clinical populations (for a meta-analysis, see^30^). In contrast, a secure attachment style is a protective factor against anxiety symptoms in healthy adolescents^31^. Although most studies have focused on the role of mother–child attachment security^28–30,32–35^, recent studies have documented the effects of paternal attachment security on psychological^36,37^ and physiological^38^ correlates of anxiety. Taken together, attachment security in children may moderate the association between individual vulnerability to anxiety and attentional bias to threat.

The primary objective of this study was to test the association between vulnerability to anxiety (as measured by a composite score grouping anxiety sensitivity, intolerance to uncertainty, and perseverative cognitions) and attentional bias to threat in healthy children. We hypothesized that greater vulnerability to anxiety would be associated with faster engagement towards threatening stimuli and slower disengagement from them. Given the prominent role of parental attachment security in conferring information about the safety of the environment, the second exploratory objective of this study was to test the moderating role of paternal and maternal attachment security on this association. We anticipated a stronger association between vulnerability to anxiety and attentional biases in children with lower attachment security, suggesting that attachment style may influence the strength of these cognitive biases.

## Material and methods

### Participants

This study, which has been approved by the ethics committee of the Centre Intégré Universitaire de Santé et de Services Sociaux de l’Est-de-l’Île-de-Montréal, is part of a larger longitudinal project designed to track the progression of anxiety symptoms in children. All methods were performed in accordance with the relevant guidelines and regulations. The current data collection represents Time 1 of this ongoing study, setting the stage for future assessments and longitudinal analyses. In total, 114 healthy children aged 8 to 12 years old (65 girls and 49 boys), with a mean age of 10.15 years, were recruited for this study from the greater Montreal area through advertisements on social media (see Sect. 3.1 for sample characteristics). Parents completed a detailed telephone interview to ensure the eligibility of their child for the study, which included questions specifically designed to screen for any history of mental health issues, including symptoms of anxiety disorders. This screening process was vital to confirm the absence of clinical anxiety and other exclusion criteria such as (i) a history of mental health problems, developmental delays, or brain damage, (ii) having a serious or unstable medical condition, and (iii) a history or current use of psychiatric medications. Given that puberty influences anxiety prevalence (for review see^39^) and emotional cognitive processes^40^, elementary school-aged children were recruited for this study. Before participating, parents provided written consent and children signed an assent form. Of note, informed consent was obtained from all subjects and/or their legal guardian(s) for the publication of identifying information/images in an online open-access publication. During the study, parents also completed some questionnaires (mostly about socio-demographic characteristics and their psychiatric symptomatology) that will not be discussed in the current manuscript. As compensation for their time, parents and children of our study were offered $10 and a $50 gift card, respectively.

### Cognitive assessment

#### Attentional bias to threat

The *Visual Search Task* validated by Williot and Blanchette^41^ was used to assess attentional bias to threat. This task was specifically selected for its effectiveness in discerning attentional engagement from disengagement processes in children^42–46^. It provides an age-appropriate, engaging format that is capable of capturing the nuanced differences in reaction times essential for identifying attentional biases^42–46^. Stimuli were presented on a Dell PC laptop computer with a standard screen (14" anti-glare FHD + WVA display with 1920 × 1200 native resolution). E-Prime software was used to present the stimuli to the participants. Responses and reaction times (RTs) to the Visual Search Task were collected from participants via designated response keys on the computer keyboard.

**Figure 1:**
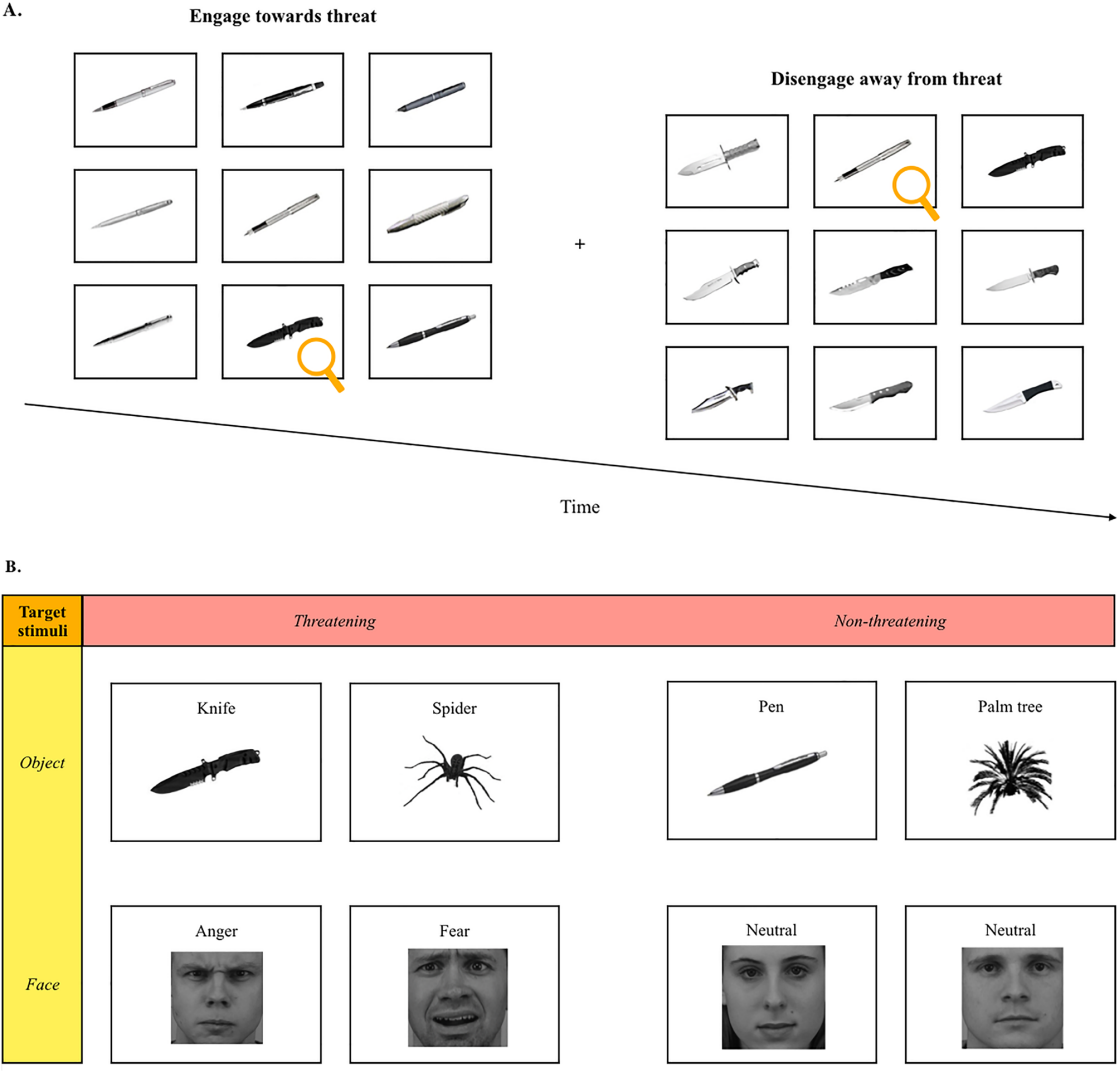
(**A**) Schematic representation of two example trials as seen by a participant. (**B**) Overview of the eight different types of target stimuli that are presented to participants. (**A**) Schematic representation of two example trials as seen by a participant. In each trial, the participant is presented with a 3 × 3 matrix. As measured by their reaction time (milliseconds), participants are asked to indicate whether the nine images belong to the same or different category by pressing different letters on a computer keyboard. In the first example trial (left matrix), the participant has to engage (processing strategy) towards the threat (knife) among eight non-threatening objects (pens). In the second example trial (right matrix), the participant has to disengage (processing strategy) away from the threat by searching for the non-threatening object (pen) among eight threatening objects (knives). For illustrative purposes, the target object (left: knife; right: pen) is indicated with an orange magnifying glass (not shown to participants during the task). (**B**) Overview of the eight different types of target stimuli that are presented to participants. Participants have to adopt a certain processing strategy (i.e., engage or disengage towards/away from threat) for specific target stimuli. The latter are classified by objects (i.e., knife, spider, pen, palm tree) or faces (i.e., anger, fear, neutral faces). For the engage towards threat processing strategy, the participant must search for the threatening object (either spiders, knives, angry faces, or fearful faces) among eight non-threatening objects (either pens, palm trees, or neutral faces). The disengage away from threat processing strategy requires the participant to search for one non-threatening object (either pens, palm trees, or neutral faces) among eight threatening objects (either knives, spiders, angry faces, or fearful faces).

At the beginning of each trial, participants were instructed to look at the fixation point (a cross) in the center of the screen. The fixation point disappeared after 500 ms and was followed by one of the matrices for the task. We used 192 different matrices, each of which contained nine images (3 × 3 matrix). This task assessed participants’ accuracy and speed in detecting specific stimuli among an array of distractors. As quickly as possible, participants were asked to determine whether the nine images belonged to the same category (by pressing the ‘A’ key on the keyboard) or to a different category (by pressing the ‘L’ key on the keyboard). As such, participants could be presented with the following types of matrices: (1) “target present trials” in which a matrix could have contained up to eight different distractors from the same category and one target image from a different category or (2) “target-absent trials” where nine different images from the same category were featured in the matrix. In the first part of the task, the stimuli were threatening and non-threatening objects. In Part 1A (“Engagement/Objects)”, participants must search for one threatening object (e.g., spider or knife) among eight non-threatening objects (e.g., palm trees or pens). These trials assess the participant’s ability to *engage* their attention toward threatening objects. In Part 1B (“Disengagement/Objects”), participants must do the opposite: search for one non-threatening object (e.g., palm trees or pens) among eight threatening objects (e.g., spiders or knives). These trials assess the participant’s ability to *disengage* their attention from threatening objects. In the second part of the task, the presented stimuli are pictures of emotional and neutral faces. In Part 2A (“Engagement/Faces”), participants must search for one emotional face (e.g., anger or fear) among eight neutral faces. In Part 2B (“Disengagement/Faces”), they must search for one neutral face among eight threatening faces (e.g., anger or fear). Similar to the first part of the task, these trials in Parts 2A and 2B assess the ability to *engage* or *disengage* their attention towards/from threatening faces, respectively (see Fig. 1A for task overview and Fig. 1B for stimuli overview). The distractors and target combinations were as follows: spiders with palm trees; knives with pens; angry with neutral faces; and fearful with neutral faces. For each part (1A: Engagement/Objects, 1B: Disengagement/Objects, 2A: Engagement/Faces, 2B: Disengagement/Faces), 24 3 × 3 matrices were presented. Each 3 × 3 matrix appeared on the screen until the participant pressed the selected key (‘A’ or ‘L’) on the computer keyboard. All targets were presented twice in the same location, randomly in one of nine possible locations, and each with different distractors. All images were presented in black and white. In addition, the brightness and contrast of the images were controlled for using the MATLAB SHINE toolkit^41^. Based on work from Williott and Blanchette (2020), all stimuli had the same orientation in each matrix. To avoid an order effect, the presentation order of the different stimuli (Faces vs. Objects) and processing strategies (Engagement vs. Disengagement) of the task was counterbalanced across participants.

### Questionnaires assessing vulnerability to anxiety

#### Childhood anxiety sensitivity index (CASI)

Children completed the validated French version of the CASI to assess their anxiety sensitivity^47^. This questionnaire features 18 items that can be answered on a 3-point Likert scale. Total scores ranged from 18 to 54. The French version of the CASI shows an internal consistency of 0.82^47^. In our sample, we found an internal consistency of α = 0.87.

#### Intolerance of uncertainty scale for children (IUSC)

We used the IUSC to assess intolerance to uncertainty^48^. Validated for children, the IUSC assesses the tendency to react negatively to uncertain situations and events at the emotional, cognitive, and behavioral levels. On a Likert scale of 1 to 5, children were asked to indicate the degree to which the 27 items describe them. Overall scores ranged from 27 to 135. Using a double-blind back-translation method, our team translated the original English version of the IUSC to French. Psychometric indices of the original version demonstrate good internal consistency (α = 0.92;^48^). In our sample, we found an internal consistency of α = 0.94.

#### Perseverative thinking questionnaire-child version (PTQ-C)

We used the French version of the PTQ-C to assess perseverative cognition in children^49^. This questionnaire consists of 15 items, where participants were asked to respond using a 5-point Likert scale ranging from 0 to 4 (0 being "never" and 4 being "almost always"). The overall score ranged from 0 to 60. A higher score indicated a greater tendency to have repeated negative thoughts. The French version of this questionnaire has good internal consistency (α = 0.90;^50^). In our sample, we documented an internal consistency of α = 0.93.

### Parent–child attachment security

#### Security scale-child self-report (SSC)

Parent–child attachment security was assessed using the validated French version of the SSC^51^. Children completed the SSC twice: once concerning their attachment relationship with their mother and once for their attachment with their father. When presented with two statements, children must choose the statement that best represents the relationship with their mother or father (e.g., “Some kids wish they were closer to their mom/dad BUT other kids are happy with how close they are to their mom/dad”) and then rate whether the selected statement was “sort of true” or “really true.” Items were scored on a 4-point Likert scale (1 = low-security level, 4 = high-security level). The security score is calculated by averaging the 15 items. The internal consistency for the French version is α = 0.76 for the mother and α = 0.82 for the father^52^. In our sample, we documented an internal consistency of α = 0.89.

### Covariate

#### Puberty development scale (PDS)

Research shows that puberty is associated with anxiety (for a review, see^39^) and emotional cognitive processes^53^. While our recruitment targeted children aged 8 to 12 to minimize the confounding effects of puberty, we administered the PDS^54^ to capture the variance in self-reported pubertal status among participants. This allowed us to control for the influence of early puberty, especially in girls, and to account for its effects in our analyses. The PDS assesses the development of secondary sexual characteristics (e.g., growth spurts, skin changes, body hair growth, breast development and menarche in girls, and voice changes and testicular growth in boys). This scale consists of five items that can be answered on a 4-point scale with an average score ranging from 0 to 4. The validated French version of the PDS has good internal consistency (α = 0.88;^55^). In our sample, we documented an internal consistency of α = 0.91.

### General protocol

#### Completion of questionnaires

Before the laboratory session, participants completed questionnaires assessing vulnerability to anxiety (CASI, IUSC, PTQ-C) and puberty (PDS) at home via Qualtrics, an online and highly secure platform. To access the questionnaires on Qualtrics, a personalized URL link was emailed to each participant’s parent(s) by a research assistant. Parent–child relationship security (SSC) was assessed at the laboratory to avoid a social desirability effect (i.e., if the questionnaire was completed at home with their parents).

#### Laboratory session

Upon their arrival at the laboratory, participants were first asked to complete the cognitive tasks (Visual Search Task and other tasks that are not mentioned in the current paper). To control for a potential order effect, the order of the tasks was counterbalanced across participants.

### Statistical analyses

Analyses were run using IBM SPSS Statistics, version 26. For the Visual Search Task, we considered average mean RTs per block (1A, 2B, 2A, 2B) for “target present” trials for correct answers only. To reduce the influence of outliers, RTs lower than 500 ms and those greater than two standard deviations above the participant’s individual mean were excluded^41^. In total, 95 children were retained in the main analyses (55 girls and 40 boys).

The distribution of our variables was also assessed for skewness and kurtosis before conducting the statistical analyses. Using indices for acceptable limits of ± 2^56^, data were found to be normally distributed. Therefore, no transformation was applied to the raw values.

#### Initial treatment of the data

Vulnerability to anxiety composite score.

Z-scores were generated for each of the following questionnaires: CASI, IUSC, and PTQ-C to create a composite anxiety vulnerability score (as used in our previous studies^24,57,58^. Then, the average of the Z-scores was calculated for each participant yielding a weighted score and was referred to as "vulnerability to anxiety."

#### Preliminary analyses

To verify whether puberty status should be included as a covariate, we conducted bivariate correlations between Puberty status (PDS score) and eight conditions of the Visual Search Task. Then, a bivariate correlation was conducted between Vulnerability to anxiety and Puberty status (PDS scores). A repeated measures ANOVA was also performed to verify whether boys and girls differed on each of these conditions. The ANOVA included Stimuli (two levels: Objects or Faces), Processing Strategy (two levels: Engagement and Disengagement), Target Type (two levels: Spider or Knives (for Objects) or Anger and Fear (for Faces)) as the within-subjects factors and Sex as a between-subjects factor. Finally, we conducted an independent samples t-test to verify whether boys and girls differed in terms of Vulnerability to anxiety. The threshold for inclusion of covariates was set at *p* < 0.100.

#### Main analyses

To verify whether Vulnerability to anxiety predicted attentional bias to threat, we conducted a repeated measures ANOVA, including the appropriate covariates (based on the results of the preliminary analyses, sex and PDS status were included as covariates). The ANOVA included Stimuli (two levels: Objects or Faces), Processing Strategy (two levels: Engagement and Disengagement), Target Type (two levels: Spider or Knives (for Objects) or Anger and Fear (for Faces)) as the within-subjects factors and Vulnerability to anxiety as a between-subjects factor, as well as all the two- and three-way interaction terms between the factors. Significant interactions were decomposed and Bonferroni corrections were applied when multiple comparisons were conducted during post hoc analyses.

To verify whether Vulnerability to anxiety and parental attachment security interacted to predict attentional bias to the threat, additional secondary analyses were conducted, including the appropriate covariates (based on the results of the preliminary analyses, sex and PDS status were included as covariates). As these analyses were based on the results of the principal analyses, they are described in the results section (see Sect. 3.3 Additional analyses: The effect of parent–child security).

## Results

### Sample characteristics

Sample characteristics are presented in Table 1.

**Table 1:** Sample Characteristics. Mean (Standard deviation). Annual income in Canadian dollars (CAD).

Sex	
Girls	65
Boys	49
Parents’ marital status (%)	
Married or in a relationship	81.96
Separated or divorced	18.04
Caucasian (%)	92.2
Annual family income (%)	
< 40 K	7.2
40 K—60 K	5.2
80 K—100 K	7.2
80 K—100 K	10.3
> 100 K	63.9
Not reported	5.2
Age	10.15 (1.19)
Anxiety sensitivity	27.00 (6.16)
Intolerance to uncertainty	53.02 (17.51)
Perseverative thinking	15.12 (9.50)
Mother–child relationship security	3.37 (0.43)
Father-child relationship security	3.26 (0.53)
Puberty	1.67 (0.66)

### Preliminary analyses

Bivariate correlations revealed significant negative associations between Puberty status (PDS score) and the four “Objects” trials: Engagement towards Spiders [r = −0.186; *p* = 0.05], Disengagement from Spiders [r = −0.308; *p* < 0.001], Engagement towards Knives [r = −0.331; *p* < 0.001], and Disengagement from Knives [r = −0.343; *p* < 0.001]. Results suggest faster RTs in children scoring higher on the PDS (i.e., being at a more advanced pubertal stage). No significant association was found with PDS for the “Faces” trials (ps > 0.493). We also found a significant association between Puberty status and Vulnerability to anxiety [r = 0.371; *p* = 0.001], suggesting accentuated vulnerability to anxiety in children with greater PDS scores.

The repeated measure ANOVA revealed no main effect of Sex (*p* = 0.379), nor two- or three-way interactions between Sex and the different parameters of the Visual Search Task (Stimuli, Target Type, or Processing Strategy; all *p* values > 0.439). Finally, the independent sample t-test revealed a sex difference [t(95) = −3.538, *p* = 0.098] with girls (M = 0.23; SE = 0.11) presenting greater vulnerability to anxiety than boys (M = −0.31; SE = 0.10).

Given the results of these preliminary analyses, Puberty status and Sex were included as covariates in the main analyses.

### Main analyses

**Figure 2:**
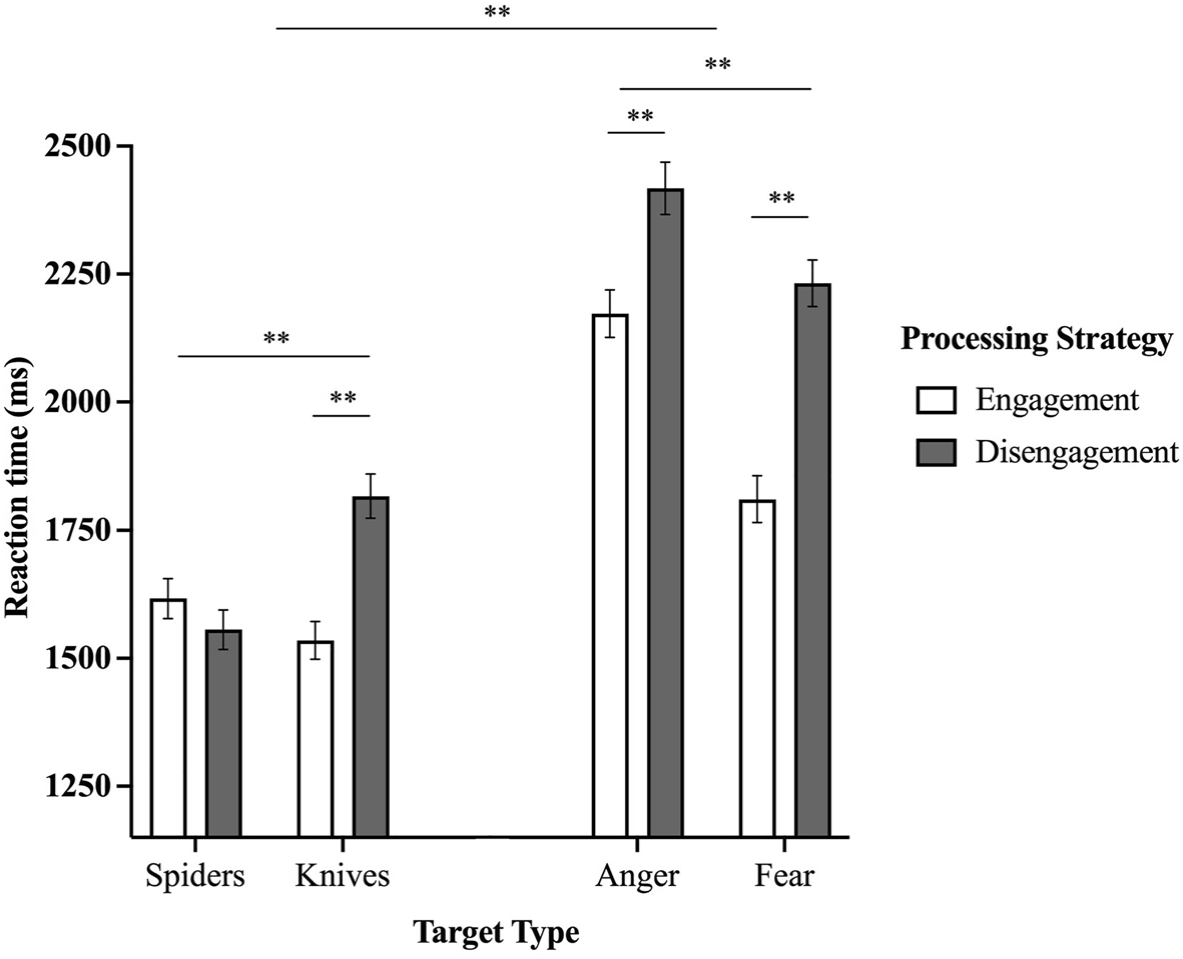
Reaction times as a function target types and processing strategies of the Visual Search Task. Means are adjusted for sex and puberty status. *p < .05; **p =  < .001; ms: milliseconds.

The repeated measures ANOVA (Fig. 2) revealed no main effect of Sex, Puberty status, or Vulnerability to anxiety (see Table 2 for statistical indices), but a significant main effect of Stimuli with faster RTs for “Objects” Stimuli (M = 1631.14, SE = 33.95) than “Faces” Stimuli (M = 2158.29; SE = 39.94). We also found a main effect of Processing Strategy, with faster RTs for Engagement trials (M = 1631.14; SE = 33.05) than Disengagement trials (M = 1783.77; SE = 33.82). We also found a Stimuli*Target Type*Processing Strategy 3-way interaction. For “Faces” Stimuli, post hoc analyses revealed faster mean RT for engaging towards angry faces (M = 2149.22; SE = 42.89) compared to disengaging from these faces (M = 2414.89; SE = 48.48), [t(95) = −5.270, *p* < 0.001]. We also found faster RTs for trials probing engagement towards fearful faces (M = 1835.37; SE = 42.20) compared to trials where the participants needed to disengage from these faces (M = 2229.63; SE = 43.57) [t(95) = −9.210, *p* < 0.001]. For “Objects” Stimuli, no differences were found for spiders as a function of processing strategy (engaging; M = 1630.28; SE = 35.76; disengaging; M = 1579.40; SE = 35.20) [t(95) = 0.700, *p* = 0.092]. However, we found faster RTs when participants had to engage their attention towards knives (M = 1552.39; SE = 35.53) versus when they had to disengage away from these stimuli (M = 1839.83; SE = 40.03), [t(95) = −9.878, *p* < 0.001].

**Table 2:** Results of the ANOVA on the reaction times of the Visual Search Task. *p = .05; **p =  < .001.

	Numerator *df*	Denominator *df*	F	*p*
Covariates				
Sex	1	91	0.966	.328
Puberty status	1	91	0.065	.800
Main effects				
Stimuli	1	91	295.318**	< .001
Target Type	1	91	21.354**	< .001
Processing Strategy	1	91	130.383**	< .001
Vulnerability	1	91	0.149	.700
Interactions				
Stimuli*Target Type	1	91	72.392**	< .001
Stimuli*Processing Strategy	1	91	29.928**	< .001
Stimuli*Vulnerability	1	91	0.925	.762
Target Type*Processing Strategy	1	91	37.221**	< .001
Target Type*Vulnerability	1	91	0.512	.476
Processing Strategy*Vulnerability	1	91	0.048	.827
Stimuli*Target Type*Processing Strategy	1	91	4.475*	.037
Target Type*Processing Strategy*Vulnerability	1	91	1.012	.317
Stimuli* Processing Strategy*Vulnerability	1	91	0.024	.887
Stimuli*Target Type*Vulnerability	1	91	5.709*	.019
Stimuli*Target Type*Processing Strategy*Vulnerability	1	91	0.167	.683

**Figure 3:**
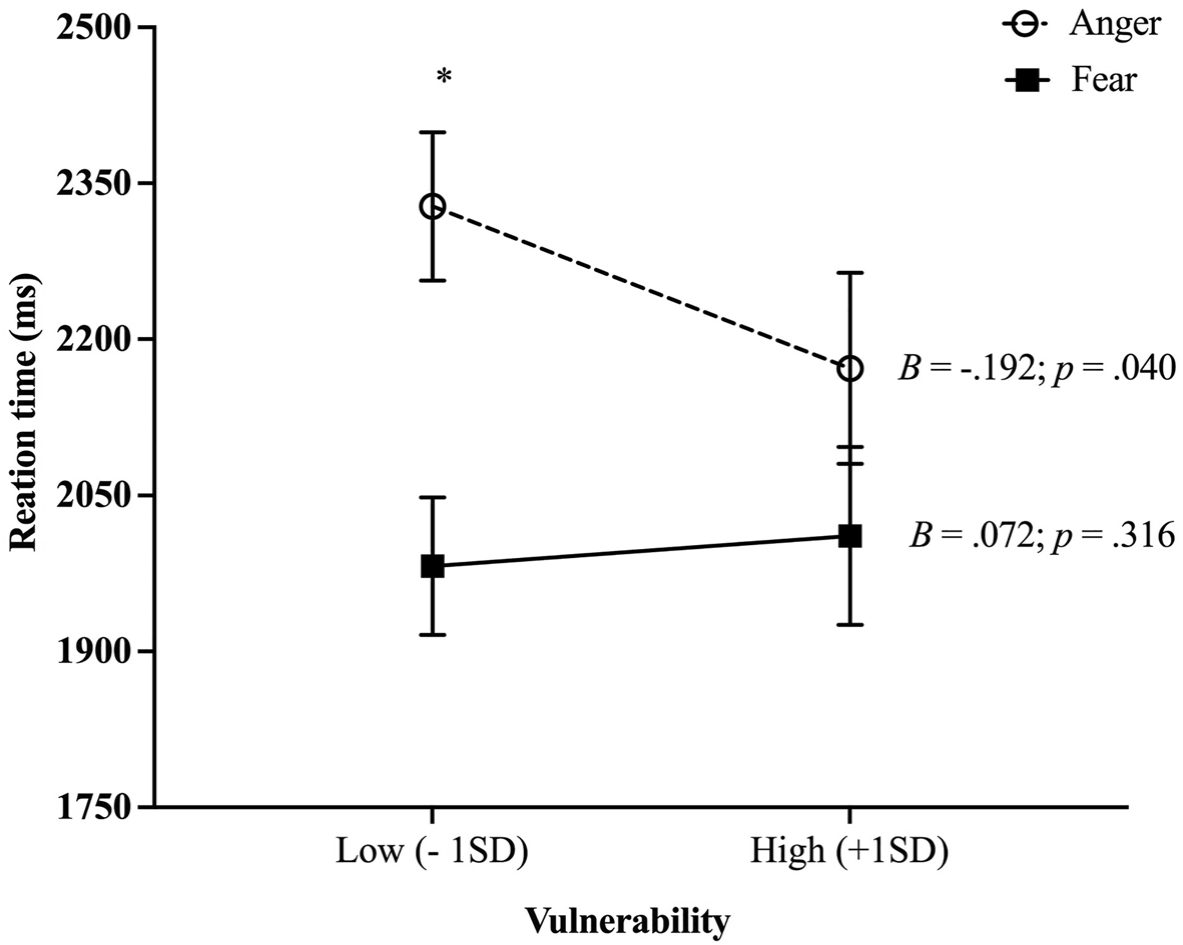
Reaction time towards Anger stimuli as a function of Vulnerability to anxiety. Means are adjusted for sex and puberty status. 1SD below and above the mean for Vulnerability to anxiety, * p < .05; ** p =  < .001; ms: milliseconds.

Finally, we found a significant Stimuli*Target Type*Vulnerability interaction. For “Faces” stimuli, post hoc tests revealed a significant Target Type*Vulnerability interaction (see Table 3A for statistical indices). Simple slope tests revealed a negative association between Vulnerability to anxiety and RTs for trials involving angry faces, irrespective of the processing strategy (i.e., engagement or disengagement; see Fig. 3 for statistical indices). Relative to children with Low Vulnerability (−1SD), children with High Vulnerability (+ 1SD) presented faster RTs towards angry faces. No effect of Vulnerability to anxiety was found for fearful faces. For “Objects”, no main or interaction effect of Vulnerability to anxiety was found (see Table 3B).

**Table 3:** Results of the post hoc analyses for Faces (A) and Objects (B) Stimuli. Averaged for both Processing Strategies (Engagement and Disengagement). *p = .05; **p =  < .001.

A. “Faces” Stimuli	Numerator *df*	Denominator *df*	F	*p*
Covariates				
Sex	1	91	0.415	.521
Puberty status	1	91	0.491	.485
Main effects				
Target Type	1	91	59.374**	< .001
Vulnerability	1	91	0.329	.569
Interaction				
Target Type*Vulnerability	1	91	4.395*	.039
B. “Objects” Stimuli	Numerator *df*	Denominator *df*	F	*p*
Covariates				
Sex	1	91	0.740	.392
Puberty status	1	91	2.194	.142
Main effects				
Target Type	1	91	4.201*	.043
Vulnerability	1	91	0.080	.778
Interaction				
Target Type*Vulnerability	1	91	0.537	.465

### Additional analyses: the effect of parent–child security

**Figure 4:**
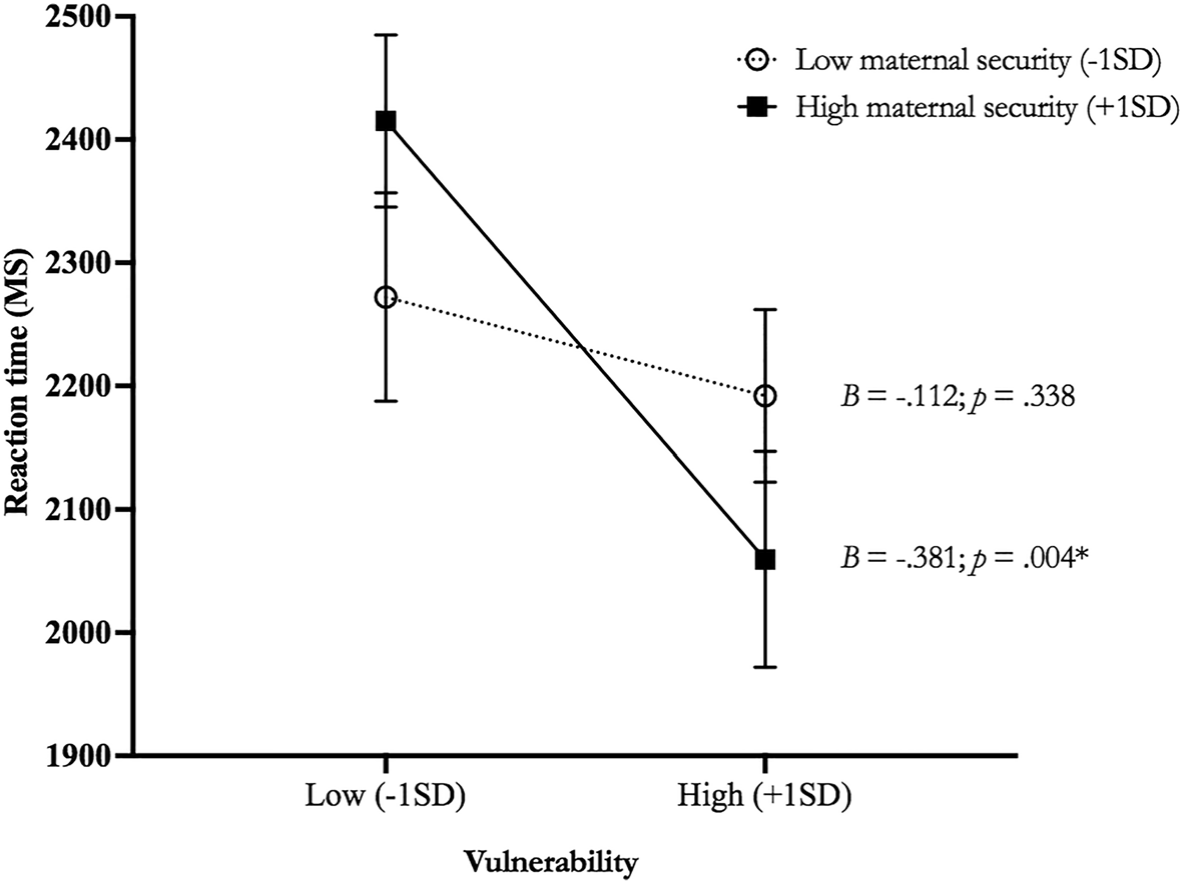
The moderating role of mother–child security on the association between vulnerability to anxiety and reaction time towards angry faces. Reaction times are averaged for both Processing Strategies (Engagement and Disengagement). Means are adjusted for sex and puberty status. 1SD below and above the mean for Vulnerability to anxiety, *p < .05. ms: milliseconds.

To better understand the factors that could account for faster RTs towards angry faces in children scoring high (+ 1SD) on Vulnerability to anxiety, we conducted additional analyses regarding parent–child relationship security. We conducted a linear regression with RTs towards Anger (mean of both Processing Strategies) as a dependent variable and Vulnerability to anxiety, Mother–child security, Father-child security, as well as the Vulnerability*Mother–child security and Vulnerability*Father-child security interaction terms as predictors. We also included Sex, Puberty Status, and RT towards fearful faces as covariates. As shown in Table 4, we found no main effect of Mother–child or Father-child security, but a Vulnerability*Mother–child security interaction was found (see Table 4 for statistical indices). Relative to children with Low Mother–child security (−1SD), children with High Mother–child security (+ 1SD) presented faster RTs towards angry faces (see Fig. 4 for statistical indices).

**Table 4:** Main and interaction effect of Vulnerability to anxiety and Mother- and Father-child security on reaction time (RT) towards Anger. *p < .05.

	Numerator *df*	Denominator *df*	F	*p*
Covariates				
Sex	1	88	0.071	.386
Puberty status	1	88	0.491	.485
RT towards Fear Stimuli		88	85.787**	< .001
Main effects				
Vulnerability	1	88	5.241*	.025
Mother–child security	1	88	0.005	.944
Father-child security		88	0.440	.509
Interaction				
Vulnerability*Mother–child security	1	88	4.076*	.027
Vulnerability*Father-child security	1	88	0.642	.426

## Discussion

The purpose of this study was to test whether patterns of attentional bias to threats (typical of clinical anxiety) were associated with vulnerability to anxiety in healthy children. Additionally, the exploratory objective of this study was to better identify the moderating role of attachment security on this association.

First, we found different reaction times as a function of the different conditions of the *Visual Search Task*. On one hand, as studies using visual search paradigms have shown quicker reaction times for faces compared to objects^59–62^, our finding that participants responded quicker to stimuli involving threatening objects (spiders and knives) at the expense of those involving threatening faces (anger and fear) is surprising. This attentional advantage for faces is thought to allow for quicker extraction of important social and emotional information from faces as it may provide a survival advantage for detecting and responding to threats in the environment^8^. As a result, the appearance of an angry or fearful face should activate threat-related processing and generate a greater attentional bias compared to less evolutionarily relevant stimuli (i.e., spiders or knives). Still, recent experimental studies have shown that an attentional bias for faces depends on stimuli features and task settings, such that certain conditions favored the detection of objects over faces^63,64^. Therefore, our results could depend on the nature of the task used, where the stimuli to be distinguished in the “Objects” conditions (e.g., knives vs. pens) were perhaps less complex compared to those in the “Faces” conditions (e.g., anger vs. neutral). On the other hand, we found that trials involving the detection of angry faces over neutral faces were responded to more quickly than those involving the detection of fearful faces over neutral faces. This finding is congruent with the literature^5,32,65^. This heightened attentional bias towards anger (compared to fear) is likely due to the different information that is conveyed by these facial expressions. While anger is a social approach-related emotion and can often indicate the presence of a potential attacker or adversary^66,67^, fear is a social-withdrawal-related emotion and can indicate the presence of a potential danger that should be avoided^68^. In consequence, the brain may prioritize processing angry faces as a means of quickly detecting and responding to potential threats in the environment^69^. Furthermore, our finding that vulnerability to anxiety predicted reaction time to angry faces was particularly novel.

Indeed, we found that vulnerability to anxiety in healthy children was associated with faster treatment of angry faces for both processing strategies, reflected by quicker reaction times for both engagement towards and disengagement from threat. Said differently, children with increased vulnerability to anxiety were rapid to engage towards and disengage away from the threat. This finding is interesting because it aligns with previous studies that have observed faster engagement toward threats in children with clinical anxiety disorders. Indeed, there is evidence for accentuated attentional engagement towards angry faces in various forms of pediatric anxiety disorders (generalized anxiety disorder, separation anxiety, and social phobias) compared to healthy populations (for a review, see^11^). However, it is crucial to distinguish between clinical and non-clinical populations. While faster engagement towards anger in healthy children might be adaptive in potentially hostile environments^70^, necessitating quick responses to threats, it may not hold the same functional value in non-hostile contexts. This raises an interesting consideration regarding the adaptability of cognitive processing in relation to environmental demands. Cognitive adaptations that are beneficial in adverse conditions may contribute to heightened vulnerability to anxiety by enhancing threat sensitivity (for a review, see^71^). Therefore, it is plausible that such adaptive mechanisms could serve a dual role: they may be protective in certain environments while simultaneously contributing to or exacerbating anxiety vulnerability in others. However, as our sample consisted of healthy children not selected based on environmental adversity, the question of whether faster engagement towards anger-related stimuli is truly adaptive remains speculative. Our study did not explicitly investigate threat processing within the context of hostile environments, hence any conclusions about the adaptability of this response pattern in our sample should be approached with caution.

Moreover, we also found that vulnerability to anxiety was associated with a faster reaction time to disengage from angry faces. This finding is the inverse of what is generally found in the literature on adult anxiety disorders. Indeed, studies found slower attentional disengagement from threat in adults suffering from generalized anxiety^72^ and anxiety-prone healthy individuals^10,73–75^. The latter findings reflect a heightened tendency for threatening stimuli to captivate selective attention^9^. An electroencephalogram study conducted on children (aged between 8 to 12 years old) showed that those with high levels of social phobia exhibited increased N2 amplitudes in response to the presentation of disgusted faces compared to their healthy counterparts^76^. This finding is noteworthy given that this neurophysiological pattern is typical of difficulties in attentional disengagement from the threat^76^. Interestingly, no behavioral differences (reaction times) were detected between groups in this study^76^. Therefore, the results obtained in the present study contrast with previous literature in adults and children suffering from anxiety disorders. One possible interpretation of the obtained results is that the processes of engagement and disengagement from threat do not follow the same developmental course with the evolution of anxiety. While hypervigilance towards the threat might appear first, difficulty in directing one's attention away from the threat may only appear once the pathology has emerged or become chronic. In sum, despite the mixed literature in anxious children and limited published work on the attentional bias to threats in healthy children, our study is one of the first to establish an association between faster engagement toward and disengagement away from threats in healthy children. Reflecting on our findings, the co-occurrence of rapid engagement towards and disengagement from threats may indicate an adaptive attentional system in healthy children. This flexibility could act as a protective factor, enabling vigilance without the risk of fixating on potential threats—a behavior linked with anxiety disorders. In contrast, children with clinical anxiety typically exhibit difficulty disengaging from threat, which could contribute to the persistence of their anxiety symptoms. Our results suggest that this attentional balance—efficient detection without excessive focus—might be a distinguishing feature between children with vulnerability to anxiety and those with clinical anxiety. Future research is warranted to explore attentional flexibility as a potential resilience factor against the development of anxiety disorders. Future studies should aim to replicate our findings and examine the potential role of additional moderating factors such as exposure to adverse life events.

Finally, secondary exploratory analyses revealed that the association between vulnerability to anxiety and faster reaction times to anger-related stimuli was more pronounced in children who had higher mother–child attachment security. This result is surprising, given that a secure parent–child relationship has often been recognized as a protective factor against the development of the anxiety disorders in children (for a meta-analysis, see^30^). This result could therefore be interpreted by the potential influence of the family environment on the child's mental health. Indeed, the family environment has repeatedly been identified as a significant risk and/or protective factor in the onset of anxiety disorders in children^77^. Various studies have demonstrated that children who grow up in households where at least one parent suffers from an internalizing disorder are two to three times more likely to develop anxiety disorders at some point in their lives^78^. One suggested explanation for this association is that the child learns anxious behaviors vicariously from the anxious parent^79^. Therefore, the role of the familial environment in shaping the child’s vulnerability to anxiety should not be minimized. Children with higher vulnerability to anxiety likely had parents who had higher expression of anxious-related traits. This being said it is possible that children who also had higher mother–child attachment security were also more synchronized with their mother and ultimately, could have prompted the expression of cognitive patterns that are more characteristic of anxiety. Indeed, while the literature on this matter is mixed, some studies have suggested a positive association between high levels of attachment security and increased dyadic synchrony^80–83^. Longitudinal follow-ups are required to specifically assess how maternal anxiety and children’s vulnerability to anxiety evolve together over time and its consequent effects on threat detection. While we propose a potential synchrony between the child's anxiety and maternal anxiety in relation to higher mother–child attachment security, we must clarify that this suggestion is strictly speculative. Our study did not directly assess parental anxiety levels, and as such, any interpretations regarding the influence of parental anxiety on the child's mental health should be approached with caution until further empirical data are available. Moreover, the moderating role of parent–child security on the association between vulnerability to anxiety and rapid threat detection was only found for mothers and their children, but not fathers. This finding may be attributable to the age of the children in our sample. As adults are exposed to a greater number of threats due to more life experience, children rely on their parents to associate negative emotions or responses to threatening stimuli^25^. Recent demographic studies showed that mothers in Western countries typically spend more time with their children than fathers, spending an average of 14.1 h per week on child care compared to fathers with 7.3 h^84^. Time spent with children and educating them about social contexts and relationships may have had a more salient impact on later threat detection in children. Similarly, as women suffer from more anxiety than men^85^ and face more socially threatening events related to anger than men (e.g., harassment, assault, marginalization;^86^), the mother–child relationship may foster rapid threat detection in children. Finally, as our sample was composed of 65 girls and 49 boys, we lacked statistical power to determine whether the sex of the child drove the mother–child attachment security moderation. Indeed, as same-sex parental figures are the most important figure in a child’s life^35^, the relationship between vulnerability to anxiety and faster response to threats may be principally moderated by high mother–child attachment security in girls.

Our study is not without its limitations. First, although we controlled for sex and puberty status, a lack of statistical power prevented us from verifying whether sex differences or puberty status influenced the association between vulnerability to anxiety and faster treatment of anger-related stimuli, as well as the moderating role of parent–child security on this relationship. Second, as this study was correlational, we were unable to draw conclusions regarding the directionality of our findings. Indeed, faster treatment of threatening stimuli may contribute to a child’s vulnerability to anxiety. Future longitudinal studies could assess attentional biases and quantify vulnerability to anxiety at several time points. To touch upon our first limitation, it would also be interesting for these longitudinal studies to be conducted over the course of several pubertal stages. Third, although we found that children vulnerable to anxiety responded quicker to threatening stimuli, it remains unclear how this affects functionality or distress. Specifically, it is important to better understand whether these quicker response times induce impairments (e.g., socially, academically) and if so, to what degree. Our research team is currently conducting a longitudinal follow-up with these children where we will be able to identify the repercussions of our findings over time. Also, we will continue to monitor the impact of puberty on the development of anxiety symptoms, enabling us to delineate the temporal relationship between vulnerability to anxiety and pubertal changes on the evolution of anxiety. Fourth, attachment security and vulnerability scores were quantified using self-report questionnaires. Studies using objective measures of parent–child attachment and vulnerability to anxiety could validate the generalizability of our findings or whether they were in part due to the self-report nature of our measures. Fifth, while our study utilized the visual search paradigm to assess attentional biases, it is one of many methodologies that can be employed for this purpose. It would be important for future research to replicate our findings using other experimental designs, such as the dot probe task or eye-tracking methodologies, to confirm and extend the understanding of attentional biases in relation to anxiety. Finally, our sample was comprised predominantly of Caucasian children from a high socioeconomic background, potentially limiting the generalizability of the findings to other populations. Future studies should aim to replicate these findings with a more diverse sample, including participants from a broader range of ethnic and socioeconomic backgrounds.

Taken together, the results of this study suggest that vulnerability to anxiety is associated with cognitive patterns that are typical of clinical anxiety. Indeed, children vulnerable to anxiety respond more quickly to stimuli involving anger than less vulnerable children. Finally, we demonstrated a preponderant role of the family environment, with a high degree of maternal security accentuating the association between vulnerability to anxiety and attentional bias to threat. In the long term, these results could contribute to the development of tools and programs for the early detection of children at risk of developing an anxiety disorder before it emerges or becomes chronic.

## Data Availability

The data that support the findings of this study are available from the corresponding author, [CR], upon reasonable request. Data are not deposited in a community-recognized repository as participants had not provided informed consent to do so.
